# Comparative Antiseizure Analysis of Diverse Natural Coumarin Derivatives in Zebrafish

**DOI:** 10.3390/ijms222111420

**Published:** 2021-10-22

**Authors:** Ewelina Kozioł, Krzysztof Jóźwiak, Barbara Budzyńska, Peter A. M. de Witte, Daniëlle Copmans, Krystyna Skalicka-Woźniak

**Affiliations:** 1 Department of Natural Products Chemistry, Medical University of Lublin, Chodzki 1, 20-093 Lublin, Poland; ewelinakoziol@umlub.pl; 2 Department of Biopharmacy, Medical University of Lublin, Chodzki 4A, 20-093 Lublin, Poland; krzysztof.jozwiak@umlub.pl; 3 Independent Laboratory of Behavioral Studies, Medical University of Lublin, 20-059 Lublin, Poland; barbarabudzynska@umlub.pl; 4 Laboratory for Molecular Biodiscovery, Department of Pharmaceutical and Pharmacological Sciences, KU Leuven, 3000 Leuven, Belgium; peter.dewitte@kuleuven.be

**Keywords:** coumarins, zebrafish, epilepsy, pentylenetetrazole, behavior, electrophysiology

## Abstract

Coumarins are a well-known group of plant secondary metabolites with various pharmacological activities, including antiseizure activity. In the search for new antiseizure drugs (ASDs) to treat epilepsy, it is yet unclear which types of coumarins are particularly interesting as a systematic analysis has not been reported. The current study performed behavioral antiseizure activity screening of 18 different coumarin derivatives in the larval zebrafish pentylenetetrazole (PTZ) model using locomotor measurements. Activity was confirmed for seven compounds, which lowered seizure-like behavior as follows: oxypeucedanin 38%, oxypeucedanin hydrate 74%, notopterol 54%, nodakenetin 29%, hyuganin C 35%, daphnoretin 65%, and pimpinellin 60%. These coumarins, together with nodakenin, underwent further antiepileptiform analysis by local field potential recordings from the zebrafish opticum tectum (midbrain). All of them, except for nodakenetin, showed pronounced antiepileptiform activity, decreasing PTZ-induced elevation in power spectral density (PSD) by 83–89% for oxypeucedanin, oxypeucedanin hydrate, and notopterol, 77% for nodakenin, 26% for nodakenetin, 65% for hyuganin C, 88% for daphnoretin, and 81% for pimpinellin. These data demonstrate the potential of diverse coumarin scaffolds for ASD discovery. Finally, the structural differences between active and inactive coumarins were investigated in silico for oxypeucedanin hydrate and byacangelicin for their interaction with GABA-transaminase, a hypothetical target.

## 1. Introduction

Epilepsy is a chronic neurological disease characterized by unprovoked recurrent epileptic seizures, affecting about 50 million people worldwide, and is still an important cause of disability and mortality today [1]. Epileptic seizures can be controlled with marketed antiseizure drugs (ASDs) in approximately 70% of patients, but, in 30% of patients, adequate seizure control is not achieved. Thus, the discovery of new ASDs, with novel mechanisms of action, is a very important area of research [2]. Pharmacological treatments using currently available ASDs have a high incidence of serious side effects including sleepiness, concentration difficulties, gastrointestinal distress, or hepatotoxicity [3]. The discovery of new drug candidates with new mechanisms of action, with less severe adverse effects and greater availability for patients, is key for future epilepsy treatment. Within the last ten years, the FDA registered 10 different drugs in the therapy of, for example, seizures in Dravet and Lennox-Gastaut syndrome and partial-onset and tonic–clonic seizures. Some of them are modifications of known structures—for example, brivaracetam based on levetiracetam, and eslicarbazepine acetate of carbazepine. Nine per 10 compounds registered in this period of time have a synthetic origin [4]. From all ASDs, only cannabidiol, gabapentine, and valproic acid (VPA) originate from natural products. VPA is an analogue of valeric acid—the natural product found in the plant *Valeriana officinalis* L. [5]. The structure of gabapentin and wigabatrine is based on the neurotransmitter γ-aminobutyric acid (GABA) [6]. Cannabidiol, approved in 2018, found in *Canabis indica* Lam and *Cannabis sativa* L., is the first drug obtained directly from plant material and seems to be safer than other ASDs [7].

Coumarins are secondary metabolites produced mainly by plants belonging to the Apiaceae family. They have attracted intense interest in recent years as a result of their diverse and potent pharmacological properties. Different studies also concluded that exposure to coumarin compounds is safe for humans [8,9]. The 2H-chromen-2-one (coumarin) heterocyclic structure has been largely studied as it is present in drugs such as warfarin and acenocumarol, which act as vitamin K antagonists, the choleretic armillarisin A, and the antibiotic novobiocin, which is a potent inhibitor of bacterial DNA gyrase [10]. Methoxypsoralen derivatives are popularly used in PUVA (psoralen and ultraviolet A) therapy in the treatment of psoriasis and vitiligo. (+)-Calanolide A, a tetracyclic dipyranocoumarin, attracted attention as a potential anti-HIV agent with activity mediated by reverse transcriptase inhibition [11,12].

Of particular interest is that coumarins also have a wide spectrum of activities on the central nervous system (CNS) thanks to their lipophilicity and the easy penetration of the blood–brain barrier [9,13]. Therefore, coumarins could be a potential valuable resource for the prevention and therapy of CNS diseases, including epilepsy. Coumarin analogs have been used as inhibitors of acetyl- and butyrylcholinesterase, monoamine oxidase, dopamine, and serotonin agonists, among others [14,15]. The antiepileptic activity of some coumarins has already been evaluated in the maximal electroshock seizure (MES) model in mice. Imperatorin, a furanocoumarin isolated from the fruits of *Angelica officinalis* L., Apiaceae, increased the seizure threshold in a dose-dependent manner [16]. This compound administered in sub-threshold doses intensified the action of classic ASDs. Similar properties have been demonstrated by xanthotoxin and the simple coumarin oshtol [9].

It is yet unclear which types of coumarins are of particular interest for ASD discovery and development as a systematic analysis of different coumarin derivatives has not been reported. Therefore, in the current study, the larval zebrafish (*Danio rerio*) pentylenetetrazole (PTZ) seizure model was used for the behavioral antiseizure activity screening of 18 coumarin derivatives, covering linear furanocoumarins, dihydrofuranocoumarins, dihydropyranocoumarins, simple coumarin derivatives, and angular furanocoumarins.

Zebrafish larvae as an animal model are suitable for screening purposes and for physiological, pathophysiological, and pharmacological analysis due to their small size and high conservation with regard to the human genome (i.e., 69% of zebrafish protein-coding genes have at least one human orthologue), targets, physiology, pharmacology, and drug metabolism [17,18,19,20]. The zebrafish model is commonly used for natural product discovery and research where material is often scarce as it only requires microgram quantities for in vivo assessment [21]; examples of recent studies are [22,23,24,25]. The exposure of zebrafish larvae to the GABA_A_ antagonist PTZ induces seizure-like behavior, which is detected by characteristic, increased locomotor activity and epileptiform activity, as revealed by electrophysiological recordings [22]. This model is now regularly used in ASD discovery studies as a good correlation in the acute PTZ test between zebrafish and rodent data was observed after the application of conventional ASDs, both on the behavioral and electrophysiological level [22].

The zebrafish-based behavioral screening approach demonstrated activity for 7 out of 18 coumarin derivatives, namely: (1) the furanocoumarins oxypeucedanin, oxypeucedanin hydrate, and notopterol, (2) the dihydrofuranocoumarin nodakenetin, (3) the dihydropyranocoumarin hyuganin C, (4) the simple coumarin derivative daphnoretin, and (5) the angular furanocoumarin pimpinellin, which was confirmed in subsequent analysis and further investigated by local field potential measurements from the optic tectum of zebrafish larvae. Moreover, the dihydrofuranocoumarin nodakenin, though inactive, was further investigated for a side-by-side comparison with nodakenetin and to understand these unique structures better as little has been reported on their biological activities so far. Finally, the possible mechanisms of discrimination between active and inactive coumarin derivatives were studied in silico, selecting oxypeucedanin hydrate and byacangelicin for their interaction with GABA-transaminase, a hypothetical target.

## 2. Results

### 2.1. Determination of Antiseizure Activity in a Zebrafish Model of Epileptic Seizures

**Figure 1:**
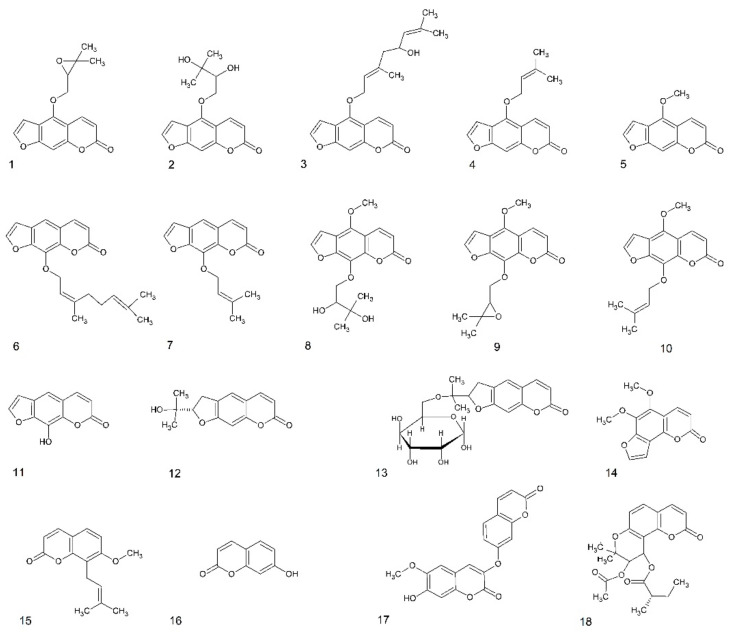
Structures of coumarins used in this study. Furanocoumarins: 1—oxypeucedanin, 2—oxypeucedanin hydrate, 3—notopterol, 4—isoimperatorin, 5—bergapten, 6—8-geranyloxypsoralen, 7—imperatorin, 8—byakangelicin, 9—byakangelicol, 10—phellopterin, 11—xanthotoxol, dihydrofuranocoumarins: 12—nodakenetin, 13—nodakenin; angular furanocoumarin: 14—pimpinellin; simple coumarin derivatives: 15—osthole, 16—umbelliferone, 17—daphnoretin; dihydropyranocoumarin: 18—hyuganin C.

The larval zebrafish PTZ seizure model was used for the behavioral antiseizure activity screening of 18 different psoralen derivatives: linear furanocoumarins, dihydrofuranocoumarins, dihydropyranocoumarin, simple coumarin derivatives, and angular furanocoumarins (Figure 1). The different coumarins were selected based on their structures and prior knowledge on their bioactivities. Eleven linear furanocoumarins were chosen because some of these structures—imperatorin, xanthotoxin, and phellopterin—have already shown activity in the MES model [9,16]. Furthermore, the dihydrofuranocoumarins nodakenin and nodakenetin were included due to the unique structures and as their CNS activity is poorly known. Finally, to gain a wide perspective of the antiseizure potential of different types of coumarins, the simple coumarin derivative daphnoretin, the angular furanocoumarin pimpinellin, and the dihydropyranocoumarin hyuganin C were also tested.

**Figure 2:**
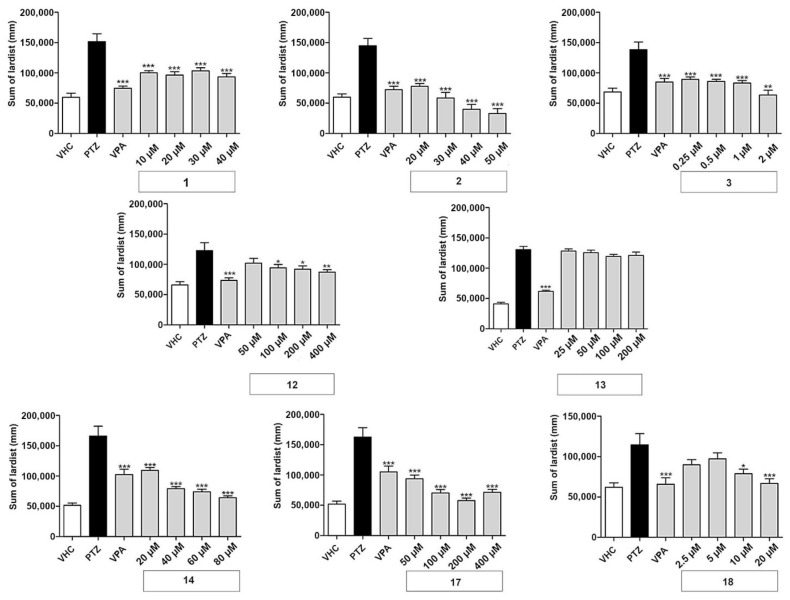
Antiseizure activity based on locomotor analysis of coumarins used in this study. PTZ-induced seizure-like behavior of larvae exposed to vehicle (VHC) only, VHC and pentylenetetrazole (PTZ), positive control valproic acid (VPA) and PTZ, or test compound and PTZ, was expressed as sum of lardist (mm) values during 30 min. Data are shown as the mean ± SEM from three independent experiments, with *n* = 10 for each experiment (*n* = 30 in total). Statistical analysis: one-way ANOVA with Dunnett’s multiple comparisons test (GraphPad Prism 5). Significance levels: * *p* < 0.05; ** *p* < 0.01; *** *p* < 0.001.

The effect of different concentrations of test compounds was explored on the locomotor behavior of larvae exposed to the GABA-antagonist PTZ. All compounds were tested at four concentrations—the maximum tolerated concentration (MTC) and two-fold, four-fold, and eight-fold dilutions of the MTC. In all experiments, larvae treated with 20 mM PTZ showed a significant increase in seizure-like behavior and locomotor activity measured as lardist sum, with an average (±SD) of 59 ± 35%. In all experiments, as expected, the positive control VPA (1 mM) decreased seizure-like behavior (i.e., PTZ-induced elevated locomotor activity) with an average (±SD) of 44 ± 34%. Moreover, 7 out of 18 coumarins tested significantly decreased PTZ-induced seizure-like behavior (Figure 2), while 11 were observed to be inactive (data not shown, except for nodakenin). The antiseizure effect depended on either the type of coumarin or the concentration. In most cases (the exception here was daphnoretin), the MTC was the most effective concentration and the lowest concentration had the weakest protective effect.

The linear furanocoumarins oxypeucedanin (1), oxypeucedanin hydrate (2), and notopterol (3) showed significant activity at all test concentrations. Oxypeucedanin (1) decreased PTZ-induced locomotor activity by 23–38%. A slight modification in structure, resulting in oxypeucedanin hydrate (2), enhanced the antiseizure effect to a concentration-dependent reduction of 43–74%. Finally, notopterol (3) lowered PTZ-induced locomotion at 0.25–1 µM to the same extent (35–39%) and by 54% at 2 µM.

In the group of dihydrofuranocoumarins, nodakenetin (12) showed significant and concentration-dependent antiseizure activity at 100–400 µM, with activities ranging between 17 and 29%, while nodakenin (13), a glycoside of nodakenetin, was inactive. Hyuganin C (18), belonging to the dihydropyranocoumarin, showed significant protective properties at 10 and 20 µM, decreasing seizure-like behavior by 27 and 35%, respectively. Daphnoretin (17), a simple coumarin derivative, significantly decreased PTZ-induced locomotor activity at all concentrations tested by 42–65%. Pimpinellin (14), an angular furanocoumarin, decreased seizure-like behavior in a concentration-dependent manner, with activities ranging from 25 to 60% reduction.

**Figure 3:**
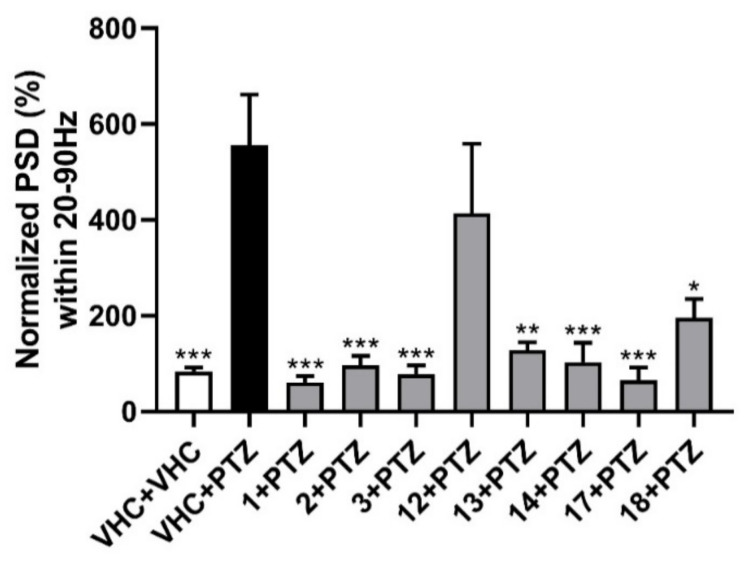
Electrophysiological antiseizure analysis of coumarins used in this study. Non-invasive local field potential recordings from the optic tectum of larvae pre-exposed to vehicle (VHC) only, VHC and pentylenetetrazole (PTZ), or test compound and PTZ. Normalized power spectral density (PSD) in defined frequency band of 20–90 Hz per individual larva is shown (mean ± SEM). Number of replicates: *n* = 9–10 for each compound group and *n* = 29–30 for the VHC + VHC and VHC + PTZ controls. Statistical analysis: one-way ANOVA with Dunnett’s multiple comparisons test (GraphPad Prism 7). Significance levels: * *p* < 0.05; ** *p* < 0.01; *** *p* < 0.001.

To further investigate the results of the behavioral study for all eight selected compounds, local field potential (LFP) measurements were employed. Zebrafish larvae were incubated with the compounds at their MTC for 18 h in darkness and the epileptiform activity was measured in the zebrafish larval optic tectum. A significant reduction in larvae with PTZ-induced epileptiform activity was observed, in comparison to PTZ-treated controls, for all compounds except nodakenetin (Figure 3).

For the rapid, objective, and automated analysis of the LFP recordings, power spectral density (PSD) analysis was performed [23]. This methodology was previously developed and used for zebrafish electrophysiology. It relies on the Welch’s method of averaging the modified periodograms with a 512-point fast Fourier transformation with 80% overlapping 100 sample (100 ms) long segments and a Hamming window [23]. PSDs were estimated for each LFP recording over each 10 Hz frequency band, ranging from 0 to 160 Hz. The results were analyzed as normalized PSD in a defined frequency band of 20–90 Hz per individual larva, as shown in Figure 3. All examined furanocoumarins at the MTC concentration ameliorated the excitatory effect of preincubation with PTZ. Oxypeucedanin (1), oxypeucedanin hydrate (2), and notopterol (3) decreased normalized PSD per larva to a similar level with 83–89%. Among the dihydrofuranocoumarins, nodakenin (13) decreased normalized PSD by 77%, while nodakenetin (12) only non-significantly reduced the PSD by 26%. Hence, while nodakenin was inactive in the locomotor assay, it has pronounced antiepileptiform activity, and the opposite is observed for nodakenetin, with antiseizure activity observed only after behavioral analysis. Pimpinellin (14, an angular furanocoumarin), daphnoretin (17, a simple coumarin derivative), and hyuganin C (18, a dihydropyranocoumarin) decreased normalized PSD by 81, 88, and 65%, respectively. Thus, except for the dihydrofuranocoumarin nodakenetin, all coumarins tested were found to be highly effective against PTZ-induced epileptiform events (electrophysiological seizures), thereby confirming their antiseizure activities as observed in the locomotor assay and, in the case of nodakenin, showing apparent antiepileptiform activity despite the absence of antiseizure activity.

### 2.2. Docking of Selected Molecules to the Structural Model of GABA-Transaminase

In order to improve our understanding of the mechanisms of discrimination between active and inactive coumarin derivatives, an in silico study of interaction for selected compounds with GABA-transaminase, a hypothetical target, was performed. The most active furanocoumarin, oxypeucedanin hydrate (2), was chosen together with an inactive coumarin from the same group, byakangelicin (8).

**Figure 4:**
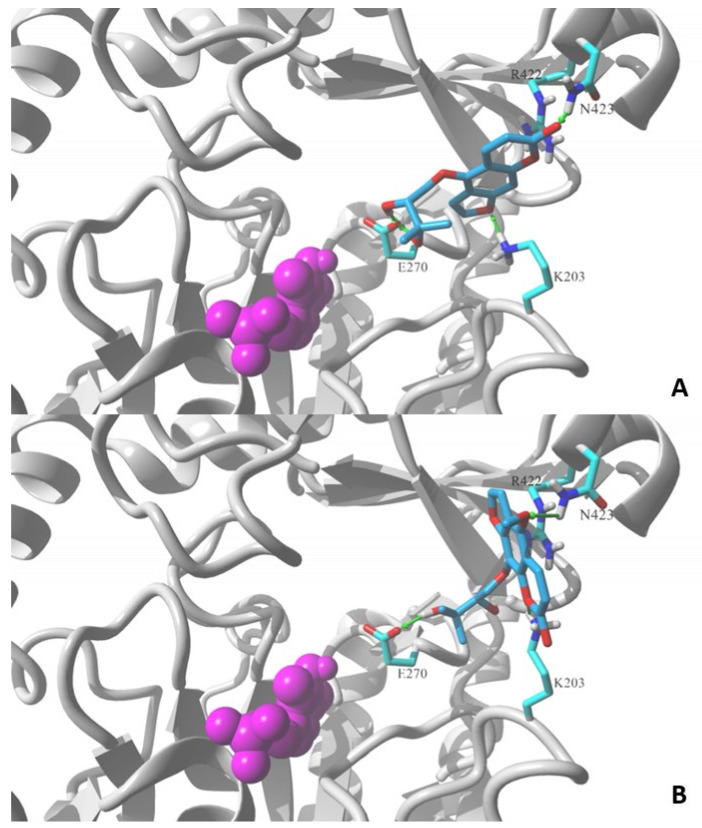
Lowest energy poses of (**A**) oxypeucedanin hydrate (2) and (**B**) byacangelicin (8) docked to a structural model of GABA-transaminase cocrystalized with pyridoxal 5′-phosphate (1OHV.pdb). Docked molecules are rendered in stick mode with atom color coded style; protein molecule is rendered in secondary structure mode (gray) and only the residues found essential for interaction with docked molecules are explicitly visualized in the stick model (discussed hydrogen bonds shown as green arrows). Pyridoxal 5′-phosphate cofactor molecule is shown in ball mode and colored in magenta. All aliphatic hydrogen atoms are hidden for clarity. Figures prepared in YASARA 19.5.23.

Figure 4 shows the lowest energy poses obtained in the docking of the two molecules using the MVD v. 6.0 simulation package. Both molecules contain a 2,3-dihydroxy-3-methylbutoxy substituent attached to the furochromenone ring either at the C5 or C8 position. Detailed comparison of obtained ligand–GABA transaminase complexes show a significant difference in the orientation of docked coumarin within the binding site. Oxypeucedanin hydrate (2), the molecule substituted at the C5 position, assumes the pose that allows hydrogen bonds between oxygen atoms of the furochromenone and polar residues at the surface of the target binding site, K203, R422, and/or N423. Additional hydrogen bonds are formed between the E270 residue and hydroxyl moieties of the substituent (Figure 4). A significantly different orientation can be observed for byakangelicin’s (8) lowest energy pose. In the case of this molecule, the arrangement where the 2,3-dihydroxy-3-methylbutoxy moiety is attached to the C8 position prevents the oxygen atoms of the furochromenone ring from forming an analogous network of hydrogen bond interactions as in the oxypeucedanin hydrate complex. As a result, the ring system is reoriented parallel to the R422 residue—an indication of possible ion–pi interaction—and K203 and N423 are capable of forming hydrogen bond interactions; however, the ring reorientation does not allow the byakangelicin molecule to form full interactions with the E270 residue. The described differences illustrate one of the possible mechanisms of discrimination between active and inactive coumarin derivatives.

## 3. Discussion

There are some literature reports about the antiepileptic activity of benzo-α-piron derivatives, especially furanocoumarins and simple coumarins. Among furanocoumarins, imperatorin, xanthotoxin, bergapten, and oxypeucedanin were tested in the maximal electroshock seizure test in mice. Imperatorin, substituted by a prenyloxy group at the C8 position, produced its maximum action at 30 min after its i.p. administration. In this case, imperatorin at doses of 50 and 100 mg/kg significantly raised the threshold for electroconvulsions in mice by 38 and 68%, respectively [16]. Xanthotoxin, structurally similar to imperatorin, but with only a methoxy group at C8, showed clear anticonvulsant activity in mice, with an ED_50_ of 219.1 ± 4.7 mg/kg, when administered 60 min before electroconvulsion [9]. On the other hand, both oxypeucedanin, with a (3,3-dimethyloxiran-2-yl)methoxy moiety at the C5 position of the psoralen ring, and bergapten (C5 substituted by methoxygroup), a structural isomer of xanthotoxin, did not show protective properties [26]; thus, substitution at C8, preferably with a long aliphatic chain, seems to be crucial for antiseizure activity.

Oxypeucedanin possesses a 3,3-dimethyloxiranat on its C5 moiety. A similar moiety with an epoxide structure is also a part of two other furanocoumarins, namely heraclenin and byakangelicol. All three compounds possess their diol forms: oxypeucedanin hydrate, heraclenol, and byakangelicin. In the study conducted by Marumoto and Miyazawa [27], all compounds (oxypeucedanin, oxypeucedanin hydrate, heraclenin, heraclenol, byakangelicin, and byakaneglicol) were compared together with 46 different coumarin structures, as β-secretase inhibitors; however, results did not suggest a direct structure–activity relationship [27]. On the other hand, both oxypeucedanin and heraclenin showed statistically significant efficiency with GABA-induced chloride currents’ potentiation [28]. Unfortunately, literature data about diol forms of furanocoumarin epoxides are limited, and, based upon our data, we hypothesize that hydrogenation enhances their antiseizure properties. Notopterol, with a much longer carbon chain than oxypeucedanin hydrate, showed activity in a more than ten times lower concentration range. Unsaturation of this carbon chain needs to be highlighted. Of note, no pharmacokinetic analysis was performed and thus differences in compound uptake might have influenced the results.

In our previous experiments, two other furanocoumarin derivatives—lucidafuranocoumarin A and bergamottin—were tested in the zebrafish epilepsy model, where antiseizure activity was based on locomotor activity analysis. Bergamottin at the most active concentration (2 µM) exhibited weak protecting potential (26% of inhibition), while lucidafuranocoumarin A (16 µM) was found to possess significant inhibition (69%) [24]. Both lucidafuranocoumarin A and bergamottin are linear furanocoumarins with a long chain in C5 of the coumarin ring. Interestingly, lucidafuranocoumarin A, similarly to oxypeucedanin, possesses an epoxy bridge in the aliphatic chain. This structural element was indicated as possibly responsible for the difference in the activity of bergamottin and lucidafuranocoumarin A.

Information about the antiepileptic activity of simple coumarins is limited. Umbelliferone (7-hydroxycoumarin) at a dose of 150 mg/kg (i.p.) 30 min before electroshock significantly increased the threshold—at around 37% [29]. Additionally, umbelliferone administrated with classic anticonvulsants—VPA and phenobarbital (PB)—enhanced the anticonvulsant effect of VPA and PB, reducing the ED_50_ from 281.4 to 215.5 mg/kg for VPA and 35.39 to 21.78 mg/kg for PB. Osthole, a simple coumarin with a methoxy group at the C7 position and an isopentenyl moiety in C8, was found to be more potent. A systematic intraperitoneal administration of the compound at 266 and 259 mg/kg, between 15 and 30 min before the test, resulted in almost 90% protection of the animals against MES [9]. In contrast to umbelliferone, osthole co-administered with carbamazepine (CBZ), PB, phenytoin (PHT), and VPA did not have a significant effect on the anticonvulsant activity of the studied drugs [29]. Both compounds were not active in the locomotor measurement assay.

In this study, we used the zebrafish PTZ seizure model and demonstrated that different coumarin derivatives show significant antiseizure activity in a behavioral assay (measuring locomotion) and significant antiepileptiform activity in an electrophysiological assay (LFP recordings). In contrast to previous studies, where a mouse model was applied, C5 substituted coumarins, oxypeucedanin, oxypeucedanin hydrate, and notopterol, showed very promising antiepileptic activity in locomotor analysis, which was confirmed with LFP recordings. In the case of two dihydrofuranocoumarins, nodakenetin and nodakenin, whose antiepileptic activities were examined for the first time, locomotor data suggested that nodakenin had no antiseizure properties, while nodakenetin did, whereas the LFP recordings showed the opposite effect, with significant antiepileptiform activity in the case of the glycoside and weak activity in the structure deprived of the glycoside moiety. Although surprising, it is not uncommon that (pronounced) activity is observed only at the behavioral or electrophysiological level [22]. Larvae incubated with dihydropyranocoumarin hyuganin C were protected against seizures at 40.2 ± 0.23%. Daphnoretin ameliorates both PTZ-induced seizure-like behavior and epileptiform activity. We can assume that the activity of daphnoretin is correlated with its structure—this derivative is a coumarin dimer and may degrade into two simple coumarins, similar to umbelliferone, which showed protective activity against maximal electroshock-induced seizures in mice. One of the most potent compounds in antiseizure protection was pimpinellin, and this is the first time that an angular furanocoumarin was examined as a potent antiseizure agent in vivo.

The antiepileptic molecular mechanism of the tested coumarins could be related to their interaction with the GABA_A_ receptor. In the study carried out by Singhuber et al. [28], the effects of 18 structurally diverse coumarin derivatives on GABA-induced chloride currents (I_GABA_) were presented. The most potent activity was shown by oxypeucedanin, osthole, pimpinellin, imperatorin, phellopterin, isoimperatorin, and heraclenin, which had a greater than 20% enhancement of I_GABA_ [28]. Thus, furanocoumarins such as isoimperatorin, imperatorin, phellopterin, and oxypeucedanin, as well as simple coumarin osthole and angular coumarin pimpinellin, showed potential to modulate the GABA_A_ receptor [28]. The highest potentiation of GABA_A_ inhibition was seen for oxypeucedanin (EC_50_ = 25 ± 8 µM, 550% of inhibition) and osthole (EC_50_ = 14 ± 1 µM, 124% of inhibition) at 100 µM. However, neither of them induced a current in the absence of GABA, which distinguishes the action of osthole and oxypeucedanin from other modulators such as etomidate or the barbiturates.

One of the molecular mechanisms used in, e.g., VPA, vigabatrin, and tiagabine is the inhibition of GABA-transaminase, which prevents GABA decomposition and increases its concentration in the brain [30]. In our study, we simulated the docking of a series of coumarin derivatives to the active center of this enzyme in order to illustrate the hypothetical mechanisms of interaction of the compounds examined in vivo. Structure–activity relationships suggest a clear discrimination of activity pattern. Coumarin derivatives containing a bulky substituent at the C5 position of the furochromenone ring, such as oxypeucedanin, oxypeucedanin hydrate, and notopterol, showed significant antiseizure activity in the locomotor assay and antiepileptiform activity in local field potential measurements. On the other hand, an analogous bulky moiety substituted at the C8 position diminishes this activity and seems to be a reason for the lack of antiseizure activity in the locomotor zebrafish assay for byakangelicin, byakangelicol, imperatorin, geranyloxypsoralen, and phellopterin. However, results obtained after the mice study [26] suggested C8 substitution in the furanocoumarin ring as crucial for antiepileptic activity, while any substitution in C5 diminishes the protective potential against seizures. For this reason, two furanocoumarins different only by the location of a fairly extensive substituent in the C5 and C8 positions were chosen for in silico analysis of the potential mechanisms of discrimination between active and inactive coumarin derivatives.

Molecular simulations allow us to hypothesize that a specific arrangement of the furochromenone ring—exposing its three oxygen atoms on the opposite side rather than a bulky substituent at C5—promotes the activity by forming optimized interactions within the binding sites of a target protein(s). In contrast, the C8-substituted ring buries these oxygen atoms on the same side as a substituent, which prevents the oxygen atoms from forming analogous interactions within the binding pocket. Such a differential pattern of molecular interactions was indeed simulated in the docking of coumarin derivatives to the active site of the GABA-aminotransferase model.

As today, the molecular target(s) responsible for the antiseizure and antiepileptiform activity of the coumarins studied here are unknown, it is yet unclear whether the GABA-transaminase target selected for our in silico study is relevant. It thus only functioned as a model to gain more insights into the molecular features associated with antiepileptic activity. In-depth mode-of-action studies will be essential to unravel the mechanisms of action of coumarins and thereby also understand their full potential for ASD discovery and development. Based upon the literature [28] and our in silico findings, interaction studies of active coumarins with the GABA_A_ receptor and GABA-transaminase would be of interest to shed more light on the possible mechanisms of antiepileptic action. Additionally, as some coumarins were reported to possess inhibitory activity against Ca^2+^ channels [31], which play a role in spreading electric impulses in neuronal tissue, they are thus also of interest for mechanistic studies.

## 4. Materials and Methods

### 4.1. Reagents

VPA and PTZ were purchased from Sigma-Aldrich (Poznań, Poland). Notopterol, oxypeucedanin, oxypeucedanin hydrate, nodakenin, and nodakenetin were purchased from Chengdu Biopurify Phytochemicals Ltd. (Chengdu, China). Byakangelicin, byakangelicol, imperatiorin, phellopterin, xanthotoxol, isoimperatorin, bergapten, osthole, 8-geranyloxypsoralen, umbelliferone, daphnoretin, and pimpinellin were purchased from PhytoLab GmbH & Co. KG (Vestenbergsgreuth, Germany). Hyuganin C was isolated by our group from *Mutellina purpurea* L. (Apiaceae) [32].

### 4.2. Experimental Animals

Adult zebrafish (*Danio rerio*) stocks of the AB strain were maintained at 28.5 °C, on a 14/10 h light/dark cycle under standard aquaculture conditions, and fertilized eggs were collected via natural spawning. Embryos were reared under standard light/dark conditions in embryo medium (1.5 mM HEPES, pH 7.1–7.3, 17.4 mM NaCl, 0.21 mM KCl, 0.12 mM MgSO_4_, 0.18 mM Ca(NO_3_)_2_, and 0.6 μM methylene blue) in an incubator at 28.5 °C. For all measurements, 7-days post-fertilization (dpf) larvae were used, which were treated at 6 dpf. The zebrafish experiments described here were approved by the Local Ethics Committee in Lublin (license no: 109/2018), the Ethics Committee of the University of Leuven (approval number 023/2017), and the Belgian Federal Department of Public Health, Food Safety & Environment (approval number LA1210199). All experiments complied with the EU Directive 2010/63/EU for animal experiments.

### 4.3. Positive Control and Proconvulsant Treatment

All compounds were dissolved in dimethyl sulfoxide (DMSO) and diluted in embryo medium to achieve a final DMSO concentration of 1% *v*/*v*. Embryo medium prepared with DMSO to a final concentration of 1% *v*/*v* served as a vehicle (VHC) control. PTZ (40 mM) was dissolved in embryo medium. The control anticonvulsant used in this study was VPA.

### 4.4. Toxicological Evaluation

The toxicity of compounds or extracts was evaluated by determining the maximum tolerated concentration, defined as the highest concentration that did not cause death and where not more than 2 out of 10 larvae exhibited any sign of locomotor impairment or loss of posture, after an 18 h incubation period [22]. Zebrafish larvae were incubated with different concentrations of tested compounds at 28.5 °C in complete darkness for 18 h. Each larva was checked under the microscope for signs of acute locomotor impairment: weak response upon a light touch of the tail with a fine needle [24], loss of posture, body deformation, exophthalmos (bulging of the eyes out of their sockets), slow or absent heartbeat, and death.

### 4.5. Evaluation of Anticonvulsant Activity

#### 4.5.1. Locomotor Analysis

Six-dpf larvae were treated with VHC (embryo medium (1% DMSO)), VPA (1 mM), or tested compound, in 100 µL volume for 18 h, in individual wells of a 96-well plate at 28.5 °C in the dark. Larvae were allowed to habituate for 5 min in a dark chamber of an automated tracking device (ZebraBox system, Viewpoint, Lyon, France). Then, 100 µL of embryo medium or 100 µL of a 40 mM PTZ solution was added to obtain a final concentration of 20 mM [33] and the locomotor activity was measured to evaluate seizure intensity. The total locomotor activity was quantified using ZebraLab software (Viewpoint, Lyon, France) [33]. Total locomotor activity was expressed as total distance moved. All tracking experiments were performed at least in triplicate.

#### 4.5.2. Electrophysiology

Non-invasive local field potential recordings were performed from the optic tectum of 7-dpf zebrafish larvae. Larvae were treated with VHC (embryo medium (1% DMSO)) or compound (MTC) in 100 µL volume (in individual wells of a 96-well plate) in darkness (28 °C) for 18 h before measurement. Prior to the recording, 100 µL of VHC (embryo medium) or 40 mM PTZ (to obtain a final concentration of 20 mM) was added to the well for 15 min. After incubation, the larva was immobilized in 2% low-melting-point agarose (Invitrogen, Carlsbad, CA, USA) and the signal electrode, an electrode inside a blunt soda-glass pipet (1412227, Hilgenberg, Germany), was positioned on the skin covering the optic tectum. Electrophysiological recordings where performed as described before [23,34]. Automated power spectral density analysis was performed using MATLABR2018b (The MathWorks, Inc., Natick, MA, USA), as described before [23,35].

### 4.6. Statistical Analysis

All statistical analyses were performed using GraphPad Prism 5 or 7 software (GraphPad Software, Inc., San Diego, CA, USA). The locomotor activity of zebrafish larvae and electrophysiological antiseizure analysis was evaluated using one-way ANOVA followed by Dunnett’s multiple comparisons test. Values were presented as lardist sum ± standard error of the mean (SEM) or normalized PSD (%) ± standard error of the mean (SEM). Lardist is large movement distance (>6 mm/s), measured in millimeters. Significance levels: * *p* ≤ 0.05, ** *p* ≤ 0.01, and *** *p* ≤ 0.001.

### 4.7. Molecular Modeling

Docking simulations were performed to the target molecular model representing pig GABA-aminotransferase cocrystalized with pyridoxal 5′-phosphate molecule (PDB id: 1OHV) [36]. Molecular models of studied compounds were constructed in HyperChem 6.05 software (Hyper Co., Gainesville, FL, USA), while the MVD v. 6.0. simulation package was used for docking simulations. In each simulation, 10 docking runs were performed, and the lowest energy pose was selected using MolDock Score function ranking. Figure 4 was prepared using the YASARA 19.5.23. package.

## 5. Conclusions

This is the first systematic investigation of the antiseizure activity of a diverse set of coumarins, including linear furanocoumarins, dihydrofuranocoumarins, dihydropyranocoumarins, simple coumarin derivatives, and angular furanocoumarins. The use of the zebrafish model allowed a combined strategy of behavioral and electrophysiological analysis and unraveled the potential of different coumarins, namely (1) the furanocoumarins oxypeucedanin, oxypeucedanin hydrate, and notopterol; (2) the dihydrofuranocoumarins nodakenin and nodakenetin; (3) the dihydropyranocoumarin hyuganin C; (4) the simple coumarin derivative daphnoretin; and (5) the angular furanocoumarin pimpinellin. The most promising new antiseizure compounds identified, which showed both antiseizure and antiepileptiform activity in the locomotor and electrophysiological assay, were oxypeucedanin, oxypeucedanin hydrate, and notopterol—C8-substituted furanocoumarins—as well as hyuganin C, daphnoretin, and pimpinellin.

## Data Availability

All important data are included in the manuscript.
